# LPS-induced systemic inflammation reveals an immunomodulatory role for the prion protein at the blood-brain interface

**DOI:** 10.1186/s12974-017-0879-5

**Published:** 2017-05-22

**Authors:** Ø. Salvesen, M. R. Reiten, A. Espenes, M. K. Bakkebø, M. A. Tranulis, C. Ersdal

**Affiliations:** Faculty of Veterinary Medicine, Norwegian University of Life Sciences, Sandnes, Norway

**Keywords:** Cellular prion protein, Systemic inflammation, Lipopolysaccharide (LPS), Innate immunity, Choroid plexus, Hippocampus, Transcriptome, Sickness behavior

## Abstract

**Background:**

The cellular prion protein (PrP^C^) is an evolutionary conserved protein abundantly expressed not only in the central nervous system but also peripherally including the immune system. A line of Norwegian dairy goats naturally devoid of PrP^C^ (*PRNP* ^Ter/Ter^) provides a novel model for studying PrP^C^ physiology.

**Methods:**

In order to explore putative roles for PrP^C^ in acute inflammatory responses, we performed a lipopolysaccharide (LPS, *Escherichia coli* O26:B6) challenge of 16 goats (8 *PRNP* ^+/+^ and 8 *PRNP* ^Ter/Ter^) and included 10 saline-treated controls (5 of each *PRNP* genotype). Clinical examinations were performed continuously, and blood samples were collected throughout the trial. Genome-wide transcription profiles of the choroid plexus, which is at the blood-brain interface, and the hippocampus were analyzed by RNA sequencing, and the same tissues were histologically evaluated.

**Results:**

All LPS-treated goats displayed clinical signs of sickness behavior, which were of significantly (*p* < 0.01) longer duration in animals without PrP^C^. In the choroid plexus, a substantial alteration of the transcriptome and activation of Iba1-positive cells were observed. This response included genotype-dependent differential expression of several genes associated with the immune response, such as *ISG15*, *CXCL12*, *CXCL14*, and acute phase proteins, among others. Activation of cytokine-responsive genes was skewed towards a more profound type I interferon response, and a less obvious type II response, in PrP^C^-deficient goats. The magnitude of gene expression in response to LPS was smaller in the hippocampus than in the choroid plexus. Resting state expression profiles revealed a few differences between the *PRNP* genotypes.

**Conclusions:**

Our data suggest that PrP^C^ acts as a modulator of certain pathways of innate immunity signaling, particularly downstream of interferons, and probably contributes to protection of vulnerable tissues against inflammatory damage.

**Electronic supplementary material:**

The online version of this article (doi:10.1186/s12974-017-0879-5) contains supplementary material, which is available to authorized users.

## Background

The cellular prion protein (PrP^C^) has been extensively studied for decades, but its normal function is still not fully understood. However, expression of this highly conserved protein across tissues in vertebrates suggests that it may have roles in a variety of physiological functions [1]. Accumulation of the misfolded isoform (PrP^Sc^) occurs in all prion disorders, and it has been postulated that loss of PrP^C^ function participates in the progression of these diseases [2]. Thus, identifying the normal function of PrP^C^ is considered an essential step in understanding the pathogenesis of prion disorders.

PrP^C^ is abundantly expressed not only in the central nervous system (CNS) but also in non-neural tissues such as gonads, the pregnant uterus, and the immune system [3–5]. Several roles for PrP^C^ in immunological processes have been suggested (reviewed in [6]). Lack of PrP^C^ seems to exacerbate inflammation, both in the periphery [7] and in the CNS [8], as well as ischemic [9–11] and traumatic [12] brain lesions. Likewise, PrP^C^ has been linked to regulation of pro- and anti-inflammatory cytokines upon systemic lipopolysaccharide (LPS) challenge [13].

Systemic administration of LPS activates the Toll-like receptor 4 signaling cascade in a range of immune cells, resulting in synthesis and release of a variety of pro-inflammatory cytokines [14]. This, in turn, induces characteristic signs of sickness behavior, which includes depression, periods of shivering, and reduced appetite and locomotor activity [15]. We recently demonstrated that LPS is a potent activator of innate immunity in goats, describing a dynamic regulation of leukocyte genes involved in immunological processes [16]. Because only small amounts of LPS and cytokines cross the blood-brain barrier (BBB) [17], information from the periphery is transmitted to the CNS through neuronal and humoral communication routes. Pro-inflammatory cytokines and LPS can stimulate the vagus nerve, directly initiating afferent signaling to the brain [18]. The humoral route is characterized by circulating cytokines that activate endothelial cells of the BBB or act on tissues that lack BBB, such as the circumventricular organs and choroid plexus (ChP) [19]. Consequently, a mirror image of peripheral cytokines is created within the brain. The ChP is localized within the brain ventricular system and is composed of vascularized stroma surrounded by a monolayer of epithelial cells. The epithelial cells are responsible for the production of cerebrospinal fluid and can release cytokines into the ventricular system. Thus, the ChP plays a key role in transmitting signals into the brain during inflammatory conditions [20, 21]. The cellular composition of the stroma can be dynamically altered through recruitment of circulating immune cells, such as lymphocytes, neutrophils, and monocytes [22, 23]. Although the hippocampus is considered more immunoprivileged than the ChP, systemic LPS challenge may also impair hippocampal function [24, 25]. Certainly, cytokine receptors such as IL1R, which is fundamental in the response to inflammatory signals, are expressed in the hippocampus [26].

Recently, a nonsense mutation early in the gene encoding PrP^C^ (*PRNP*) in Norwegian dairy goats was discovered [27]. The mutation terminates PrP^C^ synthesis only seven amino acids into the mature protein. Goats homozygous for the mutation (*PRNP* ^Ter/Ter^) are devoid of PrP^C^ and postulated to be scrapie-resistant [27, 28]. Physiological and immunological studies have not identified major disturbances under normal herd conditions, which is in agreement with studies in transgenic animals without PrP^C^ [29, 30]. However, closer phenotypic characterization indicates a small increase in red blood cell count of PrP^C^-deficient goats compared both with normal animals and with goats heterozygous for the mutation [28]. These outbred, non-transgenic goats provide a new model for studying PrP^C^ physiology.

We hypothesized that goats without PrP^C^ are more susceptible to inflammation or stressful stimuli. To investigate this, we performed a longitudinal LPS study in normal (*PRNP* ^+/+^) and PrP^C^-deficient goats (*PRNP* ^Ter/Ter^) comprising clinical, biochemical, and hematological responses, as well as end-point tissue transcriptional profiles and characterization of morphological changes. In the current paper, we focus on the PrP^C^-rich hippocampus, which is important in behavior and memory, as well as the ChP, an essential tissue in the interplay between the periphery and the brain.

## Methods

### Animals

A total of 26 Norwegian dairy goat kids, 13 *PRNP* ^Ter/Ter^ and 13 *PRNP* ^+/+^ animals, were included in the study. The goats were kept under a 16-h light/8-h dark cycle, housed in groups of two to four, and acclimatized at least 21 days before the experiment. Hay and water were provided ad libitum, and they were fed a commercial goat pellet concentrate. During the acclimatizing period, clinical examinations were performed three times, and fecal and blood samples were analyzed to ensure that the animals were healthy before the experiment. An overview of the study groups including treatment, animal number, age, weight, and gender can be found in Additional file 1a.

### LPS challenge

The goats were split in groups as follows: 16 goats (8 *PRNP* ^Ter/Ter^ and 8 *PRNP* ^*+/+*^) that received LPS intravenously and a control group of 10 goats (5 *PRNP* ^Ter/Ter^ and 5 *PRNP* ^+/+^) that were given corresponding volumes of sterile saline. Based on existing literature [31, 32] and a pilot titration study (data not included), the LPS group received a dual dose of LPS (*Escherichia coli* O26:B6, L2654 Sigma-Aldrich, USA) with a 24-h time interval between doses; 0.1 μg/kg (day 1) and 0.05 μg/kg (day 2). As goats are very sensitive to LPS, the second dosage was reduced to avoid the risk of sensitization and mortalities. The animals were euthanized by an overdose of pentobarbital 5 h after the second LPS challenge. An overview of the study protocol is given in Additional file 1b.

### Clinical examination

Clinical examination, including rectal temperature, heart and respiratory rate, and rumen contraction frequency was performed by veterinary surgeons at 12 time points during the first 7 h of day 1 and at 9 time points after the second LPS injection. Measurements of rectal temperature were repeated three times at each time point. Clinical examination was performed correspondingly, but at fewer time points, in control animals.

The clinical examination and evaluation of sickness behavior were scored blinded with respect to genotype. Signs of sickness behavior were recorded by evaluating body position (standing, lying, head and ear position), locomotor activity, social interaction, appetite, and shivering. Based on this, goats were scored as presenting “sickness behavior” (S) or “no sickness behavior” (N) every 15 min. The animals were evaluated until three consecutive “N” scorings were recorded, and the total duration of sickness behavior was calculated.

### Blood sampling, hematology and biochemistry

Blood samples (EDTA and whole blood) were drawn from *v. jugularis* using a vacutainer system (BD Company, USA). Baseline samples (0 h) were taken within 30 min before LPS challenge. The other sampling times were 1, 2, 5, and 24 h after the day 1 LPS administration. Hematology, including a complete blood count, was performed immediately by using the ADVIA 120 Hematology system (caprine analyzing program). Whole blood tubes were centrifuged, and serum stored at −20 °C until biochemical analysis. Serum total protein, albumin, and glucose were analyzed by ABX Pentra 400 (Horiba, France) and ceruloplasmin by Cobas Mira Plus (Roche). Copper was quantified by AAnalyst 300 atomic absorption spectrometer (PerkinElmer, USA).

### Histological examination

The left half of the brain was removed immediately from euthanized goats and immersion-fixed in 4% formaldehyde for 1 week. Defined brain slices were then dehydrated in graded ethanol and paraffin embedded. Morphological changes, including neuronal chromatolysis, single-cell necrosis, and inflammatory cell infiltration, were evaluated by analysis of hematoxylin and eosin-stained 4-μm-thick tissue sections. Brain regions, including hippocampus, ChP in the lateral ventricle, and obex, were investigated.

### Immunohistochemistry and semi-quantitative scoring

Paraffin sections (4 μm thick) from the abovementioned areas were mounted on Superfrost® Plus slides (Menzel-Gläser, Thermo Scientific). The distribution and morphological appearance of the astrocyte marker, GFAP (Dako, Z0334), and the microglia/macrophage marker, Iba1 (Wako, 019-19741), were investigated by immunohistochemistry. The sections were dried overnight at 58 °C, deparaffinized in xylene, and rehydrated through decreasing concentrations of graded ethanol. For Iba1 analysis, epitope retrieval was performed by trypsinization (10 mg/ml, 1:10 0.1 M Tris/HCl-buffer, 0.1% CaCl_2_) for 30 min at 37 °C. Endogenous peroxidase activity was blocked by incubation in 3% H_2_O_2_ in methanol for 10 min at room temperature. The sections were then blocked in normal goat serum (1:50) diluted in 5% bovine serum albumin (BSA) for 20 min and incubated with the primary antibodies anti-Iba1 (1.0 μg/ml) or anti-GFAP (1.9 μg/ml) for 1 h at room temperature. Further steps were performed with EnVison+ kit (Dako, K4009). The sections were counterstained with hematoxylin for 40 s. Washing between steps was in Tris-buffered saline (TBS). All runs included a negative control section where the primary antibody was replaced with 1% BSA/TBS.

The sections were examined by light microscopy and a blinded, semi-quantitative evaluation was performed by an investigator. The labeling intensity of the Iba1 and GFAP signals, the number of and localization of cells, and the appearance of primary and secondary processes were scored as follows: 0 = minimal, 1 = little, 2 = moderate, 3 = strong, including half-step grading.

### RNA extraction, quality control, and pooling

Tissue samples were collected from the right half of the brain within 15 min after euthanasia. The samples were dissected into small pieces, immediately immersed in RNAlater and stored at −80 °C. RNA extraction was carried out using RNeasy Lipid Tissue Mini Kit (Qiagen, 74804) according to the manufacturer’s instruction. The isolated RNA was quantified at optical density (OD)_260_, and purity was assessed by OD_260/280_ and OD_260/230_ absorbance readings with a DeNovix DS-11 spectrophotometer (Wilmington, USA). RNA integrity was assessed by RNA 600 Nano chips in compliance with the Agilent Bioanalyzer 2100 system in all individual samples before pooling. RNA quality data are summarized in Additional file 1c.

Extracted RNA was diluted to 500 ng/μl and then re-measured three times. Equal amounts (ng) of RNA from individual samples were pooled, reaching a final amount of 15,000 ng. The samples were pooled according to tissue, treatment, and genotype making a total of eight pools. RNA samples were shipped on dry ice to Novogene (Hong Kong) for RNA sequencing. As the transcriptome profile might be sensitive to gender, one buck (LPS, *PRNP* ^Ter/Ter^) was excluded from the material, leaving only female samples.

### RNA sequencing

After quality control, messenger RNA (mRNA) was enriched using oligo (dT) beads and then randomly fragmented. First-strand complementary DNA (cDNA) was synthesized using random hexamers and reverse transcriptase. Second-strand synthesis was done by nick-translation using a buffer containing dNTPs, RNase H, and *E. coli* polymerase I (Illumina). The cDNA fragments were processed using an end-repair reaction after the addition of a single “A” base, followed by adapter ligation. These products were then purified and amplified using PCR to generate the final cDNA library. The quality of each library was evaluated by 2100 Bioanalyzer (Agilent), followed by paired-end 150-bp sequencing on an Illumina HiSeq2000. The quality control summary can be found in Additional file 1d.

### Differential expression analysis

Raw reads (FASTQ) were clipped and trimmed of adapter contamination, and those of low quality were removed. Quality-controlled FASTQ files were mapped to the *Capra hircus* (domestic goat) reference genome using the TopHat2 (v2.0.12) software with two mismatches. Mapping status is summarized in Additional file 1e. Differential gene expression analysis (DEA) was performed using DEGSeq2 (1.12.0) with the following criteria: Log2 ratio ±0.59 (fold change ±1.5) and a false discovery rate (FDR) adjusted *q*-value (*q* < 0.05). For each tissue, four DEAs were performed. Differences in basal transcriptome levels were assessed by comparing *PRNP* ^Ter/Ter^ (saline) to *PRNP* ^+/+^ (saline). The genomic response to LPS in each genotype was assessed by comparing the LPS groups with the saline-treated control of the matching genotype. Finally, DEAs between *PRNP* ^Ter/Ter^ (LPS) and *PRNP* ^*+/+*^ (LPS) were performed to identify differences between the genotypes during acute inflammation. FPKM (fragments per kilobase of exon per million fragments mapped) values, which take into account the effects of both sequencing depth and gene length, were used to estimate gene expression levels. Genes encoding ribosomal subunit proteins are not included in the tables.

### Gene ontology enrichment analysis

To characterize the overall LPS effect, gene ontology (GO) analysis was performed on genes that were differentially expressed (DEGs) in at least one of the *PRNP* genotypes. We used the online PANTHER classification system to identify over-represented biological processes among the DEGs [33, 34]. Because the *C. hircus* genome was not available, and the *Bos taurus* genome resulted in fewer mapped genes, the well-annotated *Homo sapiens* genome was used as reference. The fold enrichment displays the over-representation of genes in a given biological process, compared with the expected number in the reference genome. *p* values <0.05 represents a statistical significant over-representation and are calculated by the binomial test as described in [33]. In total, eight genes (*SAA3*, *OAS1L*, *MHCI*, *IFI203*, *VCAM*, *ADGRG6*, *C4*, and *C21H14orf132*) were not mapped to the GO reference genome.

### Validation of RNA sequencing by qPCR

First, 600 ng total RNA from each individual sample was converted into first-strand cDNA using QuantiTect Reverse Transcription Kit (Qiagen, Germany) according to the manufacturer’s instructions. A non-reverse transcriptase control (NoRT) and no template control (NTC) were included.

The expression of *PRNP*, *IFI6*, *CXCL10*, and *SAA3* genes was investigated by Light cycler 480 qPCR using SYBR Green PCR Master Mix under the following conditions: initial denaturation for 5 min at 95 °C, followed by 40 amplification cycles (10 s at 95 °C, 10 s at 60 °C, and 15 s at 72 °C) and construction of melting curves. For each primer assay, a pool of cDNA samples was used to make three separate series with the following dilutions: 1:2, 1:10, 1:50, 1:250, and 1:1250. Standard curves were constructed to obtain primer amplification efficiencies, correlations, and dynamic range. Internal normalization was performed against the *ACTB* reference gene, and relative expression was calculated using the 2^−ΔΔCq^ method [35]. Primer sequences are given in Additional file 1f.

### Descriptive and statistical analyses

Clinical, biochemical, hematological, and qPCR expression data are presented as mean ± standard error of the mean (SEM). Graphical and statistical analyses were performed in GraphPad Prism 6 (GraphPad software Inc., USA) and Microsoft Excel 2013. Comparisons between two groups were performed using Student’s *t* test, assuming equal variance.

## Results

### Prolonged sickness behavior in goats devoid of PrP^C^

**Fig. 1:**
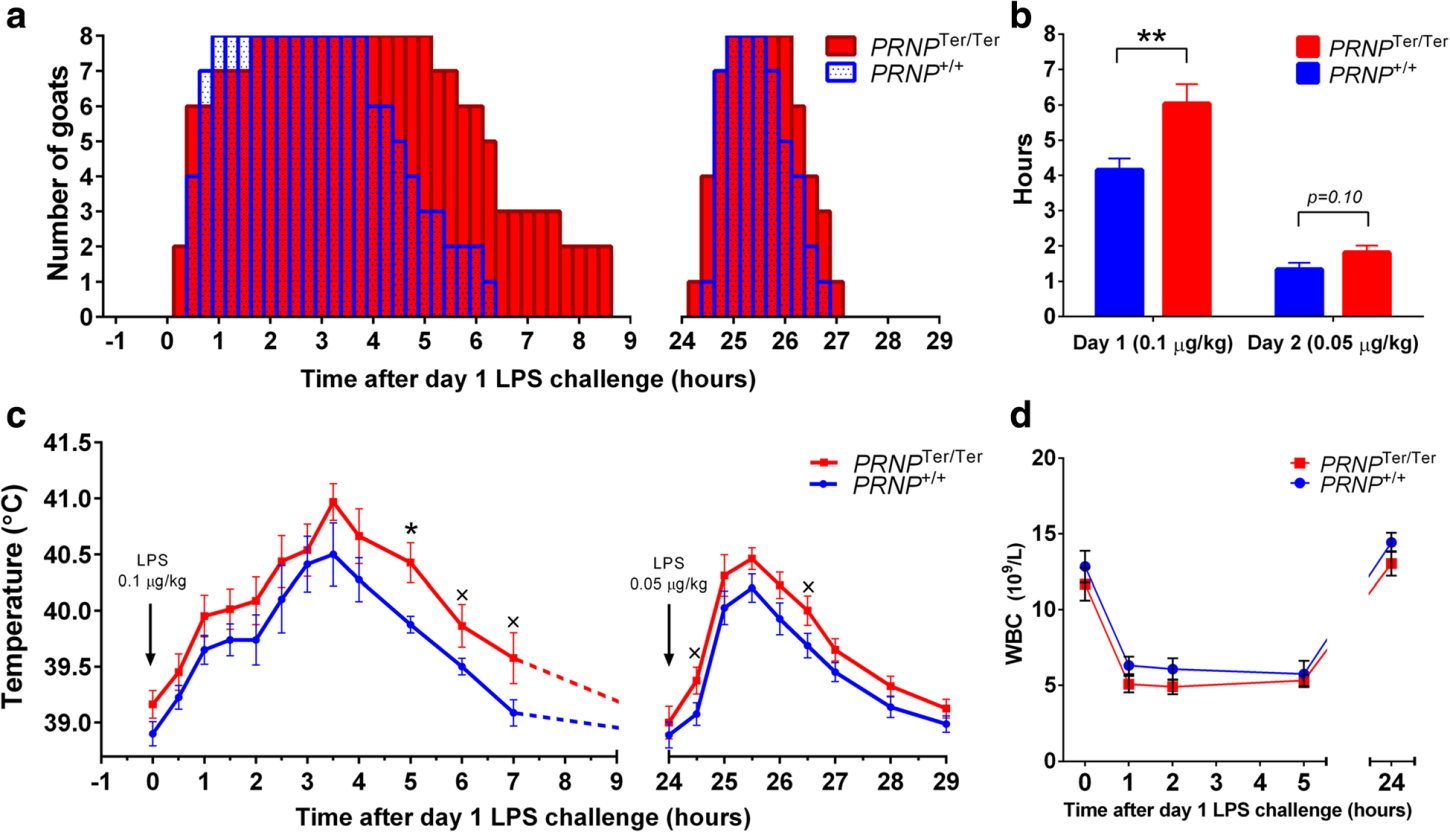
Sickness behavior, rectal temperature, and white blood cells after LPS challenge. Goats were suspected to a dual LPS challenge; 0.1 and 0.05 μg/kg. The diagrams show the number of goats displaying sickness behavior at the indicated time points (**a**) and the mean duration of sickness behavior after LPS challenge (**b**). The rectal temperature (**c**) and total number of white blood cells (**d**) are shown at the indicated time points after LPS challenge. Values are mean ± SEM, *n* = 16. **p < 0.01; *p < 0.05; ^×^ *p* < 0.1 (trend)

Within the first 2 hours after LPS administration, all 16 goats displayed characteristic signs of sickness behavior, such as lowered head, hanging ears, and periods of shivering, as well as reduced social interaction, appetite, and locomotor activity. The mean duration of sickness behavior was significantly (*p* < 0.01) longer in the *PRNP* ^Ter/Ter^ group (6.03 ± 0.59 h), than in the *PRNP* ^+/+^ goats (4.16 ± 0.33 h) after day 1 LPS challenge (0.1 μg/kg). The second LPS injection (0.05 μg/kg) induced a significantly shorter period of sickness behavior compared with day 1, but the difference between the genotypes was non-significant (Fig. 1a, b). *PRNP* ^Ter/Ter^ goats displayed a slightly higher mean body temperature at all time points throughout the experiment. The fever response was biphasic, with the lower peak at 1.5 h and the highest temperature at 3.5 h on day 1. A quick onset monophasic fever, peaking at 1.5 h was measured on day 2 (Fig. 1c). Tachycardia was observed in both genotypes, reaching a maximum at 5 h post challenge (data not shown).

The number of white blood cells declined after LPS challenge (Fig. 1d), abruptly in neutrophils and monocytes and more gradually in lymphocytes, with no differences between the genotypes (Additional file 2a). This contrasts with what has been reported in *Prnp*-knock out (KO) mice following systemic LPS injection [13], and our data do not support a role for PrP^C^ in leukocyte extravasation and recovery. Total serum protein decreased in both groups, and the level of albumin was significantly lower in *PRNP* ^Ter/Ter^ animals at 5 and 24 h. No differences were observed in serum ceruloplasmin or copper. Although impaired glucose homeostasis has been observed in *Prnp*-KO mice [36], blood glucose was regulated similarly between the two genotypes after LPS challenge (Additional file 2b). None of the control animals displayed alterations in clinical, hematological, or biochemical parameters in response to saline injection and handling stress.

### Systemic LPS challenge induces substantial alterations in the choroid plexus transcriptome and reveals differences between *PRNP* genotypes

**Fig. 2:**
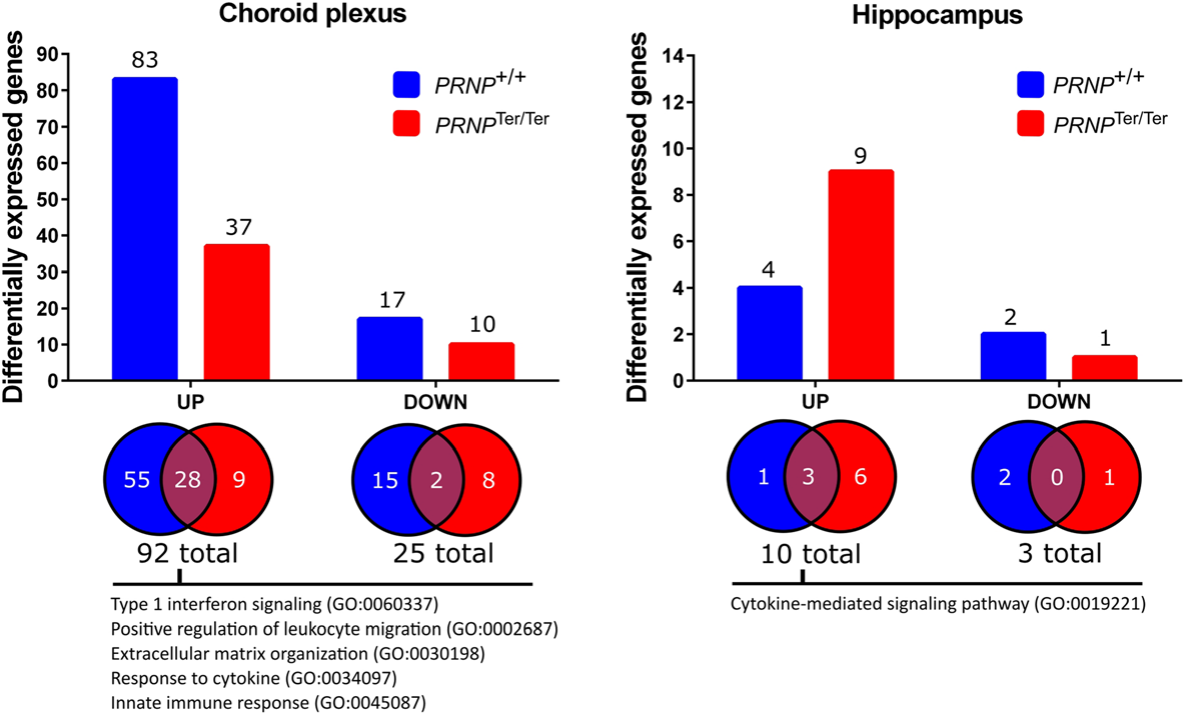
Upregulated and downregulated genes in the choroid plexus and hippocampus after LPS challenge. Differential expression analyses were performed by comparing the LPS groups to the saline-treated control group of the matching genotype (log2 ratio ±0.59 and *q* < 0.05). Venn diagrams show genes that overlap in the two genotypes. The top over-represented biological functions (GO terms) are included for upregulated genes. LPS, *n* = 15. Saline, *n* = 10

In the ChP, 92 genes were upregulated and 25 genes downregulated in at least one *PRNP* genotype upon LPS challenge (Fig. 2). Eighty-seven percent of the DEGs were regulated in the same direction in both *PRNP* genotypes, but some had a log2 ratio or *q*-value outside our filtration criteria (Additional file 3). GO enrichment analysis of upregulated genes displayed an over-representation of genes involved in type I interferon (IFN) signaling, collagen catabolism, and leukocyte migration. Additionally, 19 genes were characterized as being involved in the innate immune response and 23 genes were cytokine-responsive (Additional file 4a).

**Fig. 3:**
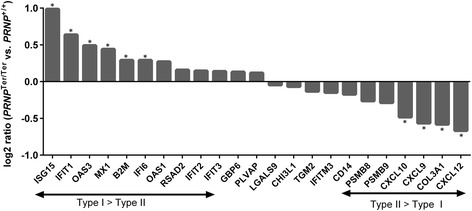
Comparison of ChP cytokine-responsive genes (GO:0034097) after LPS challenge between genotypes. Expression levels were investigated by transcriptome analysis (Illumina HiSeq 2000) on RNA extracted from the choroid plexus. Transcripts of genes stimulated primarily by type I interferons increased in PrP^C^-deficient goats compared with *PRNP* ^+/+^ goats, whereas genes stimulated primarily by type II interferons were reduced. *Fold change ±1.2 and *p* < 0.05. *n* = 15

Finally, we identified 25 differentially expressed genes between the two genotypes after LPS treatment, which included several immune genes, as well as genes involved in extracellular matrix stability and oxidative phosphorylation (Table 1). Given the previous report on cytokines being influenced by PrP^C^ [13], we analyzed the 23 genes characterized as cytokine-responsive (GO:0034097) with less stringent criteria (fold change ±1.2 and *p* < 0.05). Comparing the log2 ratio of the these genes after LPS administration, a relatively higher type I IFN response, and less prominent type II IFN response, was noted in the PrP^C^-deficient goats (Fig. 3).

**Table 1:** Choroid plexus DEGs between *PRNP* genotypes after LPS treatment

Gene ID	Symbol	Gene name	Log2 ratio	Top functions
100860813	MHC II	HA25	1.80	Antigen presentation (extracellular pathway)
102178155	FTSJ1	FtsJ RNA methyltransferase homolog 1	1.53	Methyltransferase
102169982	ISG15	ISG15 ubiquitin-like modifier	0.99	ISGylation, innate immunity
102189650	ANK3	Ankyrin 3	0.75	Cell motility, activation, proliferation,
102187800	UBB	Ubiquitin B	0.73	Targeting of proteins for degradation
102184593	SPOCK2	SPARC/osteonectin, cwcv and kazal-like domains proteoglycan 2	0.68	Extracellular matrix structure
102183219	BGN	Biglycan	−0.60	Extracellular matrix structure, innate immunity
102188061	ATP5E	ATP synthase, H+ transporting, mitochondrial F1 complex, epsilon subunit	−0.63	Catalyzes ATP synthesis, oxidative phosphorylation
102185420	COL24A1	Collagen type XXIV alpha 1 chain	−0.66	Extracellular matrix structure
102172487	CP	Ceruloplasmin	−0.66	Acute phase protein, ferroxidase enzyme
102169556	CXCL12	C-X-C motif chemokine ligand 12	−0.67	Chemoattractant, innate immunity
102172637	LECT1	Leukocyte cell derived chemotaxin 1	−0.69	Promotes chondrocyte growth, inhibit angiogenesis
102170107	ATP5J2	ATP synthase, H+ transporting, mitochondrial Fo complex subunit F2	−0.70	Catalyzes ATP synthesis, oxidative phosphorylation
102181355	PCOLCE	Procollagen C-endopeptidase enhancer	−0.75	Collagen precursor peptidase activator
100860756	OGN	Osteoglycin	−0.76	Growth factor activity
102179198	TNC	Tenascin C	−0.81	Extracellular matrix structure
102182694	CXCL14	C-X-C motif chemokine ligand 14	−0.81	Chemoattractant, immunomodulatory
102177419	NDUFA1	NADH:ubiquinone oxidoreductase subunit A1	−0.85	Component of the respiratory chain, mitochondria
102191086	COCH	Cochlin	−1.03	Extracellular matrix, pro-inflammatory, cytokine regulatory
102187830	COL17A1	Collagen type XVII alpha 1 chain	−1.04	Hemidesmosome component
102189939	ATP5I	ATP synthase, H+ transporting, mitochondrial Fo complex subunit E	−1.58	Catalyze ATP synthesis, oxidative phosphorylation
100860915	ASIP	Agouti signaling protein	−2.14	Paracrine signaling, pigmentation
102180584	MHC I	BOLA class I histocompatibility antigen, alpha chain BL3-7	−2.32	Antigen presentation (cytosolic pathway)
102169975	PRNP	Prion protein	−2.80	Unknown, cytoprotective
102176354	HP	Haptoglobin	−3.25	Acute phase protein, bind hemoglobin

Differential expression analysis was performed by comparing LPS-treated *PRNP* ^Ter/Ter^ vs. *PRNP* ^+/+^ (log2 ratio ± 0.59 and *q* < 0.05). *n* = 15

### Systemic LPS challenge induces minor alterations in hippocampus transcriptome in both *PRNP* genotypes

According to the filtration criteria, only 10 genes were upregulated and 3 genes downregulated, in one or both *PRNP* genotypes after LPS challenge (Fig. 2 and Table 2). Five of the upregulated genes (*MT2*, *CXCL9*, *CXCL10*, *TGM2*, and *IFI6*) were classified as being involved in cytokine signaling (Additional file 4b). *CXCL10*, *CXCL9*, and *TGM2* were significantly upregulated in both genotypes, whereas IFI6 was only upregulated in normal goats. The expression of IFI6, however, was already high at rest in *PRNP* ^Ter/Ter^ goats (saline group). Six genes (*ATP5I*, *GFAP*, *HOPX*, *MT2*, *MT1A*, and *SLC14A1*) were significantly upregulated in the PrP^C^-deficient goats and slightly, but non-significantly, upregulated in the normal goats. In the LPS-treated normal goats, a slight increase in *PRNP* expression was observed by RNA sequencing and qPCR, but this was not statistically significant.

**Table 2:** Upregulated and downregulated genes in the hippocampus after LPS challenge

			Log2 ratio, LPS vs. saline		
Gene ID	Symbol	Gene name	*PRNP* ^+/+^	*PRNP* ^Ter/Ter^	Top functions
102185230	IFI6	Interferon alpha inducible protein 6	1.19*	−0.05	Regulation of apoptosis
100860873	CXCL10	C-X-C motif chemokine ligand 10	3.37*	3.07*	Chemoattractant
102187851	CXCL9	C-X-C motif chemokine ligand 9	2.93*	2.65*	Chemoattractant
102185477	TGM2	Transglutaminase 2	1.25*	1.36*	Unclear, involved in phagocytosis
102189939	ATP5I	ATP synthase, H+ transporting, mitochondrial Fo complex subunit E	0.53	0.76*	Catalyze ATP synthesis
102190069	GFAP	Glial fibrillary acidic protein	0.34	0.62*	Cell communication, mitosis, BBB function
102178715	HOPX	HOP homeobox	0.35	0.65*	Unknown
102188072	MT2	Metallothionein-2	0.29	0.91*	Metal-binding, neuroprotection
102188618	MT1A	Metallothionein-1A	0.58	1.40*	Metal-binding, neuroprotection
100860878	SLC14A1	Solute carrier family 14 member 1	0.54	1.59*	Membrane transport (urea)
102186073	AQP4	Aquaporin 4	−0.61*	−0.31	Membrane transport (water)
102175716	MYO10	Myosin X	−0.66*	−0.53	Motor molecule, bind actin
102186825	COL9A2	Collagen type IX alpha 2 chain	−0.43	−0.64*	Extracellular matrix structure

Differential expression analyses were performed by comparing the LPS groups to the saline control group of the matching genotype. Italic values indicate a true differential expression. LPS, *n* = 15. Saline, *n* = 10

*log2 ratio ±0.59, *q* < 0.05

### Transcriptome analyses of choroid plexus and hippocampus at rest (saline) reveal minor differences between the *PRNP* genotypes

The basal expression was investigated by a differential expression analysis of the saline-treated groups, and only minor differences were observed. *PRNP* ^Ter/Ter^ goats displayed higher expression of IFI6 in both hippocampus and in the ChP. *DKK3*, *CHGA*, and *MYOM2* were slightly upregulated in the PrP^C^-deficient goats, whereas the transcript levels of *PRNP* were decreased with a log2 ratio of −4.4 in the hippocampus and −2.3 in the ChP. However, as RNA was extracted from tissues as a whole, the dilution effect might mask more subtle phenotypes related to loss of PrP^C^ in certain cell populations.

### Systemic LPS activates Iba1-positive cells in the choroid plexus and astrocytes in the hippocampus in both *PRNP* genotypes

**Fig. 4:**
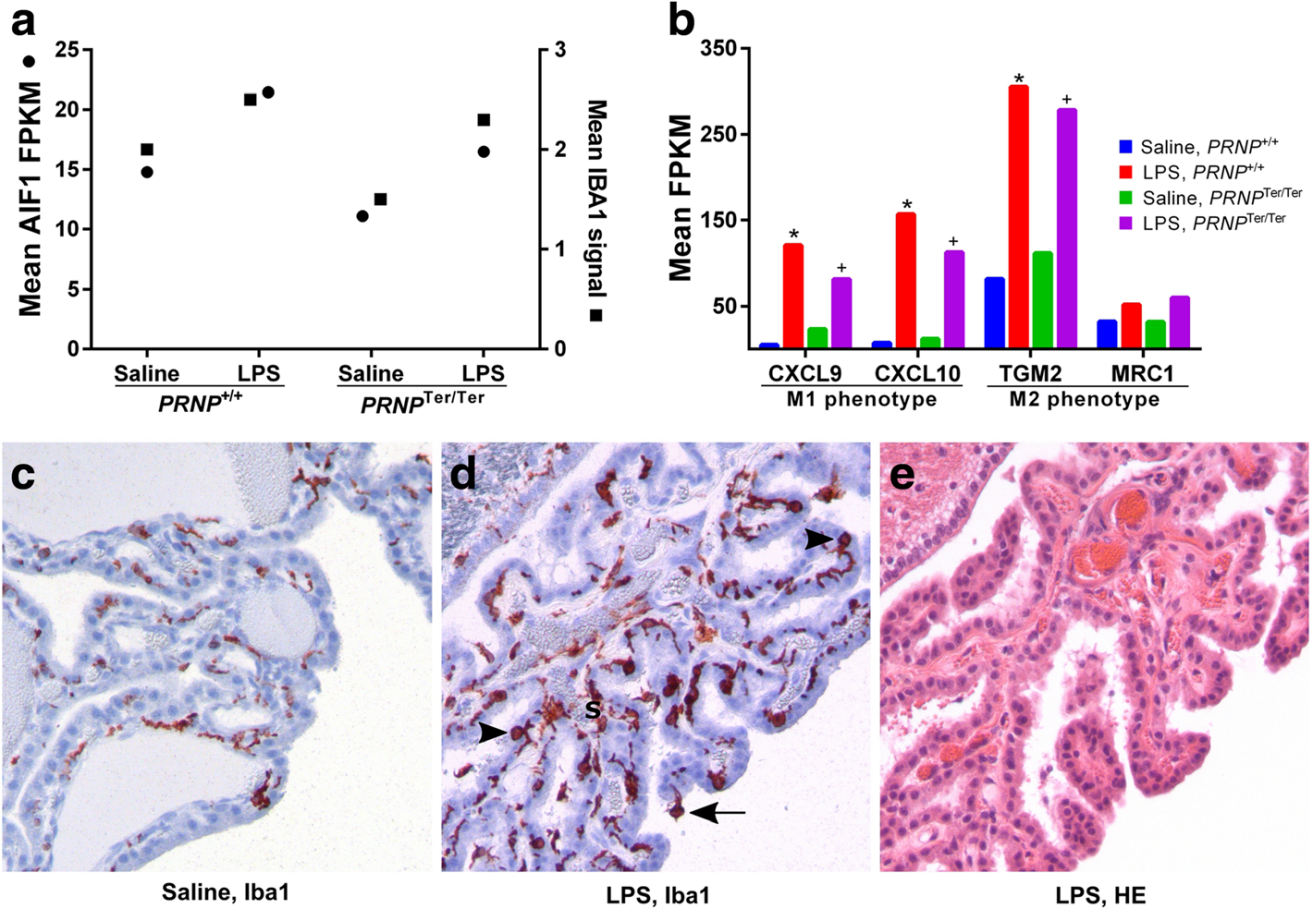
LPS upregulates AIF1/IBA1 and macrophage/microglia phenotype markers in the choroid plexus. Expression levels were investigated by transcriptome analysis (Illumina HiSeq 2000) on RNA extracted from the choroid plexus. Allograft inflammatory factor 1 (*AIF1*) expression corresponded with increased IBA1 signal detected by immunohistochemistry (**a**). Genes indicating activation of macrophage/microglia phenotype M1 and M2 increased in both *PRNP* genotypes. A significant upregulation (log2 ratio >0.59, *q* < 0.05) in *PRNP* ^+/+^ and *PRNP* ^Ter/Ter^ goats is indicated by an *asterisk* and *plus sign*, respectively (**b**). Some Iba1-positive cells are present in the choroid plexus of saline-treated animals (**c**). The number of Iba1-positive cells is increased, and the cells have a different location and longer processes in LPS-treated animals. Cells are localized within the stroma (*S*), between the epithelial cells (*arrowhead*) and protruding from the apical surface (*arrow*) (**d**). No infiltration of inflammatory cells is observed within the stroma (**e**). ×200 magnification. LPS, *n* = 16. Saline, *n* = 10

In the ChP, expression of the Iba1-encoding allograft inflammatory factor 1 (*AIF1*) and of the microglia/macrophage phenotype activation markers was increased (Fig. 4a, b). This corresponded with increased signal and number of Iba1-positive cells (Fig. 4c, d). These cells were primarily located at the basal side of the epithelial cells and within the stroma, with processes extending between the epithelium and around blood vessels. Some Iba1-positive cells were presumably migrating towards the apical surface and found between the epithelial cells and at the apical surface. No GFAP-labeling was observed in the ChP, confirming that this tissue does not contain astrocytes. Evaluation of the HE-stained sections did not reveal infiltration of inflammatory cells within the stroma (Fig. 4e), but an increased number of leukocytes was observed in the blood vessels (leukostasis).

**Fig. 5:**
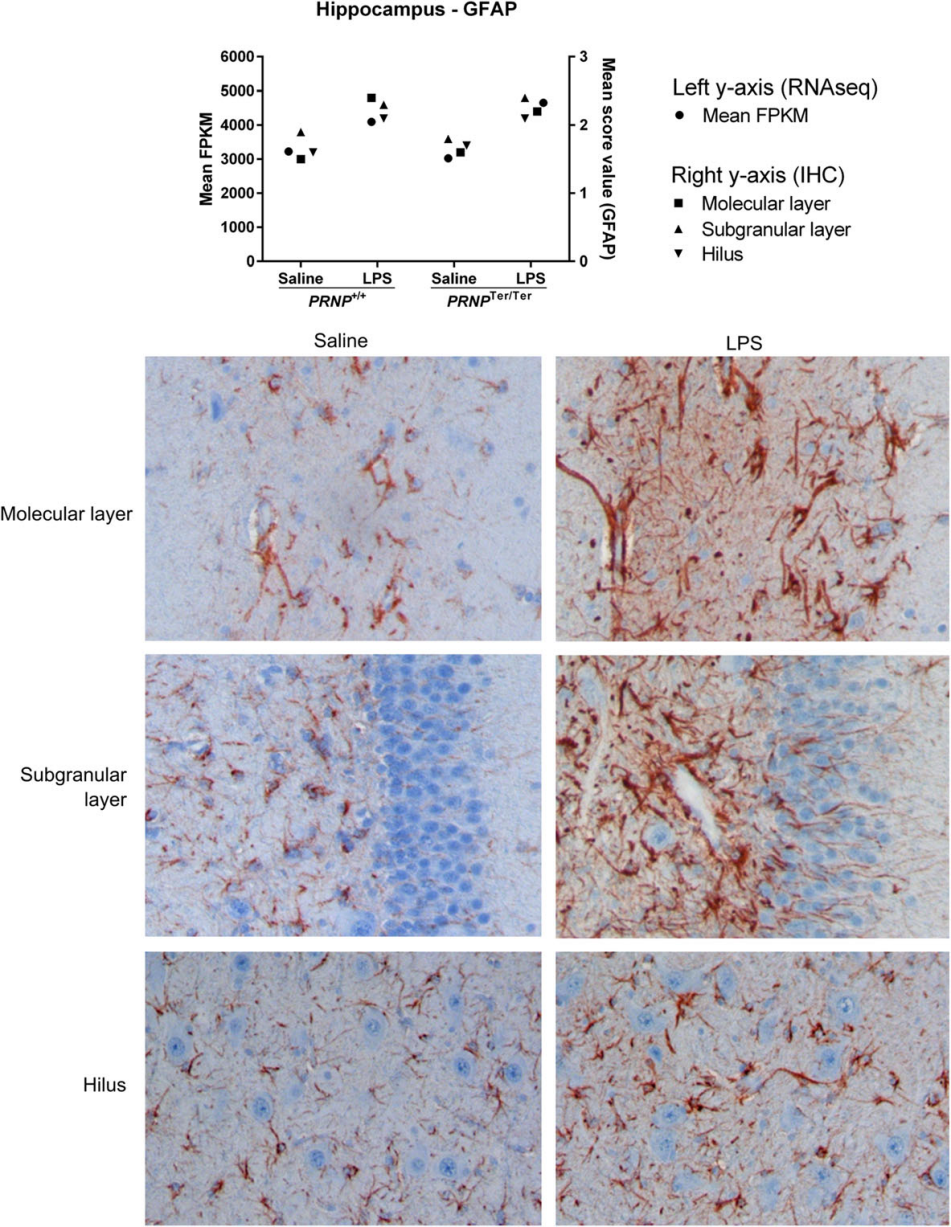
LPS upregulates *GFAP* expression and GFAP signal in hippocampus in both *PRNP* genotypes. Mean *GFAP* expression was investigated by transcriptome analysis (Illumina HiSeq 2000) on RNA extracted from the hippocampus. GFAP immunohistochemistry (IHC) shows increased GFAP signal in the molecular layer, subgranular layer, and hilus after LPS administration. Activated astrocytes have more distinct primary and secondary processes. ×200 magnification. LPS, *n* = 16. Saline, *n* = 10

In the hippocampus, LPS treatment upregulated *GFAP* expression, and increased GFAP signal was detected by immunohistochemistry in the molecular layer, subgranular layer, and hilus (Fig. 5). Activated astrocytes had more distinct primary and secondary processes, than seen in saline controls. Morphological evaluation of Iba1-stained microglia did not identify any effect of treatment or genotype in the hippocampus or obex. Some individuals had a few single-cell necrosis in the granular and subgranular layer, but this was not related to genotype or treatment. No evidence of disruption of the BBB was observed by light microscopy.

### Validation of RNA sequencing by qPCR on individual RNA samples

Four target genes (*PRNP*, *IFI6*, *CXCL10*, and *SAA3*) were investigated by qPCR on individual RNA samples from both tissues. A strong correlation (*r* = 0.989, *p* < 0.0001, Pearson correlation) was observed between expression values of RNAseq and qPCR, and differential expression was confirmed in 26 out of 28 comparisons. The increased IFI6 expression in *PRNP* ^Ter/Ter^ goats at rest was primarily due to large biological variability. In the hippocampus, *SAA3* was not detected by RNAseq in saline groups, and comparison with relative qPCR expression was not possible (Additional file 5).

## Discussion

The high degree of conservation of the *PRNP* gene across species [37] suggests that the protein possesses important biological functions. These have, however, proven difficult to pin-point even after the creation of *Prnp*-KO mice [38]. It has been proposed that compensatory mechanisms could mask loss-of-function phenotypes under normal conditions and become evident during stress such as inflammation. For instance, *Prnp*-KO mice displayed an exacerbated disease progression of experimental autoimmune encephalomyelitis [8] and colitis [7]. Here, we report the first study of the inflammatory response in a unique, non-rodent model naturally devoid of PrP^C^. Considering the high *PRNP* expression in the hippocampus and the role of the choroid plexus (ChP) in responding to inflammatory signals at the blood-brain boundary, we investigated both these tissues by full-scale transcriptome analysis. Our data suggest a role for PrP^C^ in modulating the innate immune response.

Systemic LPS challenge induced characteristic signs of sickness behavior that was prolonged by about 2 h in *PRNP* ^Ter/Ter^ goats after the initial high dose of LPS (0.1 μg/kg). This is a novel clinical loss-of-function phenotype, pointing to a more potent inflammatory response in the absence of PrP^C^. When the dosage was halved on day 2, the mean duration of sickness behavior was only about 1–2 h in both groups. The difference between genotypes was similar as day 1, but not statistically significant (*p* = 0.1). This suggests that the lower dose of LPS did not induce a sufficient amount of inflammatory stress to clearly separate the two genotypes. In the ChP, a clear acute phase response as well as activation of a range of interferon-stimulated genes was observed, which underlines the widespread role of these genes in the host defense to bacterial endotoxin [16]. Interestingly, several genes associated with the immune response were differentially expressed between the *PRNP* genotypes after LPS challenge. This included acute phase proteins genes and multiple chemokines, as well as *COCH* that has an anti-bacterial role by regulating local cytokine production [39]. Based on previous reports of PrP^C^ regulating cytokines [13], we compared the 23 genes characterized as cytokine-responsive (GO:0034097) between the two genotypes. Most of these genes are primarily induced by type I and/or type II IFNs [40, 41]. Notably, there was a relatively more pronounced type I IFN response and a weaker type II response in PrP^C^-deficient goats, compared with the normal group. A potential role of PrP^C^ in regulating type II IFN response has been previously suggested, as IFN-γ levels were decreased in ConA-treated PrP 0/0 splenocytes [42], but type I interferon signaling has received less attention. Type I interferons are key modulators of innate immunity and may affect the manifestation of sickness behavior by facilitating the immune activation of other cytokines [43]. Indeed, interferon signaling is involved in many of the effects previously attributed PrP^C^, such as apoptosis [44], protection against oxidative stress [45], DNA repair [46], and depressive-like behavior immediately after stress [47, 48]. Taken together, our data indicate that PrP^C^ contributes as a modulator of innate immunity signaling, particularly downstream of type I interferons, which might affect the duration of sickness behavior.

The substantial activation of the ChP transcriptome, including upregulation of *AIF1* expressed by activated macrophages/microglia, corresponded with a parallel increase in Iba1 signal. Markers of classical activation M1 (*CXCL9*, *CXCL10*) and alternative activation M2 (*TGM2*, *MRC1*) increased [49, 50], suggesting a combination of M1 and M2 phenotype of activated macrophages/Iba1-positive cells. Moreover, cytokines involved in leukocyte migration were upregulated, as well as genes involved in collagen catabolism and extracellular matrix organization, indicating that the integrity of the blood-cerebrospinal fluid barrier was altered. These findings agree with the observation of increased numbers of Iba1-positive cells in the ChP stroma, some of which were presumably migrating through the epithelium. The stromal cells could represent antigen-presenting cells as dendritic cells [51], recently blood-derived monocytes, or residing macrophages [52].

Not surprisingly, alterations in the hippocampus transcriptome were modest compared with those observed in the ChP, yet a somewhat similar cytokine response was present. However, it is possible that the filtration of single genes strictly by fold change and *q*-value might exclude biologically relevant pathways characterized by a subtle increase in a subset of genes. The two most upregulated genes in the hippocampus were *CXCL9* and *CXCL10*, which are primarily induced by IFN-γ signaling [41]. Recently, *CXCL10* expression in hippocampus was traced to activated astrocytes and cells lining the blood vessels [53]. This suggests that endothelial cells within the BBB, as well as nearby glial cells, react to circulating LPS and cytokines by releasing IFN-γ, which, in turn, stimulates expression of *CXCL9* and *CXCL10*. Despite the important role of these chemokines in recruiting immune cells into the brain [54], no inflammatory cell infiltration was observed in our study. Although the overall LPS response in the hippocampus was similar in the two *PRNP* genotypes, two metallothioneins (MT) were significantly upregulated in PrP^C^-deficient goats. Metallothioneins bind metals and scavenge free radicals and participate in reducing the inflammatory and oxidative stress [55]. In the brain, MT-I and MT-II are primarily expressed by activated astrocytes [55]. We further found that *GFAP* transcription increased significantly in *PRNP* ^Ter/Ter^ goats, indicating an early activation of astrocytes as previously described [53, 56]. This was confirmed by an increased GFAP labeling after LPS treatment. Given the role of MTs [55] and PrP^C^ [57] in neuroprotection, it is tempting to speculate that upregulation of metallothioneins in astrocytes could be part of a compensatory mechanism in goats devoid of PrP^C^.

Systemic administration of LPS has been shown to activate microglia in the hypothalamus, thalamus, and brainstem as early as 8–24 h after LPS challenge [58], but murine hippocampal microglia were not activated until 48 h post challenge [59]. The latter study is consistent with our results as we did not observe increased AIF1 expression or altered Iba1 immunohistochemical labeling in hippocampus 29 h after the first LPS injection. Altogether, the transcriptional and morphological findings indicate that only a modest inflammation, with a predominance of astrocytes, was present in the hippocampus. This might not be sufficient to manifest clearly potential phenotypes related to the loss of PrP^C^ and further suggests that this brain region is relatively protected from circulating endotoxins. Still, the clinical signs of sickness behavior, and difference between the *PRNP* genotypes in this respect, demonstrate the sensitivity of the CNS towards inflammatory insult and that this sensitivity is increased in the absence of PrP^C^.

Although not statistically significant, LPS upregulated *PRNP* transcripts in both the hippocampus and ChP of *PRNP* ^+/+^ goats, indicating a role for PrP^C^ in acute inflammation. Similarly, systemic LPS upregulated PrP^C^ in circulating neutrophils [60], whereas LPS incubation increased *PRNP* expression in neuronal cell cultures [61]. As expected, *PRNP* expression was low in PrP^C^-deficient goats, regardless of treatment, which probably reflects nonsense-mediated mRNA decay [62].

## Conclusions

This is the first report of an endotoxin challenge in a non-transgenic goat model naturally devoid of PrP^C^. Animals without PrP^C^ suffered a significantly prolonged period of sickness behavior after LPS challenge. Transcriptome data revealed that in the absence of PrP^C^, LPS induced an increased expression of a number of genes downstream of type I interferons. These results, together with the finding that *PRNP* was slightly upregulated upon LPS stimulation in normal goats, point to an immunomodulatory role for PrP^C^ during inflammation. Importantly, a huge number of proteins contribute to modulating inflammatory responses, balancing pro- and anti-inflammatory signaling. This balancing act is vitally important for the organism and not dependent upon a few proteins or signaling pathways. Considering the many crossroads between innate immunity signaling and various aspects of cellular homeostasis, such as apoptosis and DNA repair, our data contribute to a new understanding of cellular functions previously ascribed to PrP^C^ and provide directions for future mechanistic studies.

## Additional files

Additional file 1: Material and methods. Study groups, experimental protocol, RNA quality control, RNA sequencing quality control and mapping status, and primer sequences. (PDF 395 kb)

Additional file 2: Hematology and biochemistry after LPS challenge. (PDF 590 kb)

Additional file 3: List of upregulated and downregulated genes in choroid plexus after LPS challenge. (PDF 458 kb)

Additional file 4: Gene ontology analyses of DEGs after LPS challenge. (PDF 312 kb)

Additional file 5: Validation of RNAseq data by qPCR. (PDF 484 kb)

## Abbreviations

ChP: Choroid plexus
CNS: Central nervous system
DEA: Differential expressed analysis
DEGs: Differentially expressed genes
GO: Gene ontology
IL1R: Interleukin 1 receptor
INF: Interferon
LPS: Lipopolysaccharide
NoRT: No reverse transcriptase
NTC: No template control
qPCR: Quantitative real-time polymerase chain reaction
RIN: RNA integrity number
