# Supplementary material for: LPS-induced systemic inflammation reveals an immunomodulatory role for the prion protein at the blood-brain interface
Source: J Neuroinflammation. 2017 May 22;14:106. doi: 10.1186/s12974-017-0879-5 (PMC5441080; doi:10.1186/s12974-017-0879-5)
Supplement: Supplementary file 1 — Material and methods. Study groups, experimental protocol, RNA quality control, RNA sequencing quality control and mapping status, and primer sequences. (PDF 395 kb) [file 12974_2017_879_MOESM1_ESM.pdf]

Additional file 1 – Material and methods

a) Study groups showing treatment, animal number, mean age, mean weight and sex.

Treatment	Genotype	Animal number	Mean age (months)	Mean weight (kg)	Sex
**LPS**	*PRNP*$^{+/+}$	8	7,3 (±0,3)	27,3 (±2,7)	8♀
	PRNP$^{Ter/Ter}$	8	6,7 (±1,1)	26,1 (±4,6)	7♀, 1♂
**Saline**	*PRNP*$^{+/+}$	5	6,7 (±0,2)	21,5 (±3,2)	5♀
	PRNP$^{Ter/Ter}$	5	6,6 (±0,03)	24,9 (±2,8)	5♀
		**26**	**6,9 (± 0,7)**	**25,3 (± 3,9)**	**25♀, 1♂**

b) Experimental protocol. The LPS-group (8 *PRNP*$^{Ter/Ter}$ and 8 *PRNP*$^{+/+}$*)* received a dual dose of LPS with a 24 hour time interval between doses; 0.1 µg/kg (day 1) and 0.05 µg/kg (day 2). A control group of 10 goats (5 *PRNP*$^{Ter/Ter}$ and 5 *PRNP*$^{+/+}$) received corresponding volumes of sterile saline. Blood samples (EDTA and whole blood) were taken at 0 h (baseline), and at 1 h, 2 h, 5 h and 24 h. Goats were euthanized 5 h after day 2 LPS challenge, and tissue samples for RNA sequencing and histology/IHC were collected.

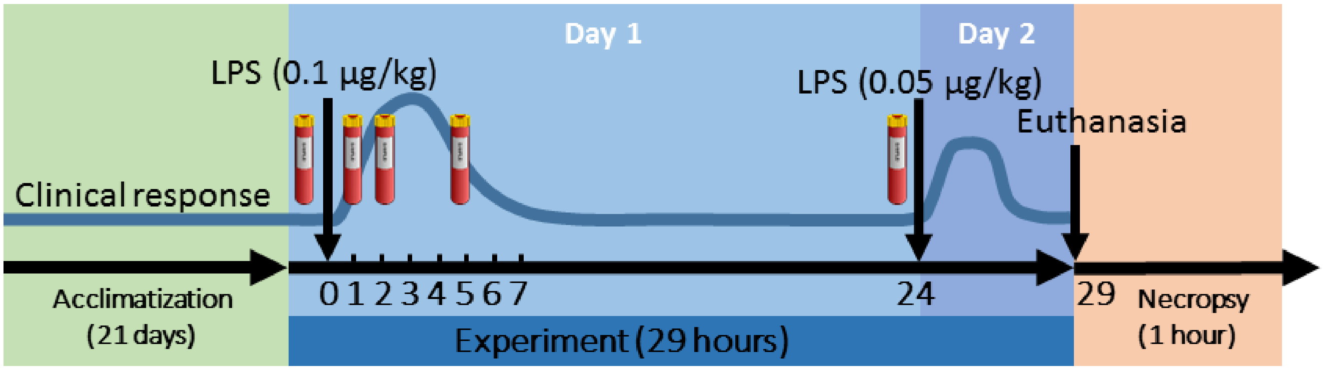

c) RNA quality control. Purity was based on $OD_{260/280}$ and $OD_{260/230}$ absorbance reading using DeNovix DS-11 spectrophotometer (Wilmington, USA). RNA integrity was assessed by Agilent Bioanalyzer system.

Tissue	Treatment	Group	$OD_{260/280}$	$OD_{260/230}$	Mean RIN	Novogene RIN*
**Hippocampus**	**LPS**	*PRNP*$^{Ter/Ter}$	2.1 (±0.14)	2.2 (±0.07)	7.4 (±0.32)	7.9
		PRNP$^{+/+}$	2.1 (±0.07)	2.1 (±0.07)	6.9 (±0.49)	7.3
	**Saline**	*PRNP*$^{Ter/Ter}$	2.1 (±0.16)	2.2 (±0.11)	7.0 (±0.23)	7.3
		PRNP$^{+/+}$	2.1 (±0.10)	2.2 (±0.09)	6.7 (±0.44)	7.1
**Plexus choroideus**	**LPS**	*PRNP*$^{Ter/Ter}$	2.1 (±0.06)	2.1 (±0.14)	8.5 (±0.15)	9.1
		PRNP$^{+/+}$	2.1 (±0.04)	2.2 (±0.05)	8.5 (±0.23)	9.1
	**Saline**	*PRNP*$^{Ter/Ter}$	2.1 (±0.03)	2.2 (±0.03)	8.6 (±0.11)	8.9
		PRNP$^{+/+}$	2.1 (±0.07)	2.1 (±0.18)	8.3 (±0.66)	8.7

* Novogene quality control on pooled samples before RNA sequencing. All samples were assessed as being of the best quality level (A level).

d) RNA sequencing - quality control summary

Sample	Raw reads	Clean reads	clean bases	Error rate (%)	Q20 (%)	Q30 (%)	GC content (%)
Hi_L_Ter	29491364	29011306	4.35G	0.01	98.13	95.56	50.29
Hi_L_Nrm	44613392	43879676	6.58G	0.01	97.98	95.26	49.86
Hi_C_Ter	36437780	35798088	5.37G	0.01	97.97	95.27	50.46
Hi_C_Nrm	33616850	33064478	4.96G	0.01	97.97	95.22	50.16
Pl_L_Ter	32970148	32247800	4.84G	0.01	97.77	94.76	51.22
Pl_L_Nrm	36319188	35355340	5.3G	0.01	98.24	95.77	50.71
Pl_C_Ter	36164222	35219560	5.28G	0.01	98.22	95.71	50.61
Pl_C_Nrm	34593166	33672136	5.05G	0.01	98.13	95.52	50.17

e) RNA sequencing - overview of mapping status

Sample	Hi_L_Ter	Hi_L_Nrm	Hi_C_Ter	Hi_C_Nrm	Pl_L_Ter	Pl_L_Nrm	Pl_C_Ter	Pl_C_Nrm
Total reads	29011306	43879676	35798088	33064478	32247800	35355340	35219560	33672136
Total mapped	22835125 (78.71%)	34333597 (78.24%)	27539126 (76.93%)	25520388 (77.18%)	25094428 (77.82%)	28358931 (80.21%)	28505198 (80.94%)	27394726 (81.36%)
Multiple mapped	445358 (1.54%)	731596 (1.67%)	553167 (1.55%)	546319 (1.65%)	521416 (1.62%)	554083 (1.57%)	523055 (1.49%)	580696 (1.72%)
Uniquely mapped	22389767 (77.18%)	33602001 (76.58%)	26985959 (75.38%)	24974069 (75.53%)	24573012 (76.2%)	27804848 (78.64%)	27982143 (79.45%)	26814030 (79.63%)
Read-1	11467892 (39.53%)	17262764 (39.34%)	13842319 (38.67%)	12847045 (38.85%)	12699443 (39.38%)	14219284 (40.22%)	14331584 (40.69%)	13764042 (40.88%)
Read-2	10921875 (37.65%)	16339237 (37.24%)	13143640 (36.72%)	12127024 (36.68%)	11873569 (36.82%)	13585564 (38.43%)	13650559 (38.76%)	13049988 (38.76%)
Reads map to '+'	11176520 (38.52%)	16779682 (38.24%)	13457666 (37.59%)	12446395 (37.64%)	12266866 (38.04%)	13892065 (39.29%)	13977428 (39.69%)	13390416 (39.77%)
Reads map to '-'	11213247 (38.65%)	16822319 (38.34%)	13528293 (37.79%)	12527674 (37.89%)	12306146 (38.16%)	13912783 (39.35%)	14004715 (39.76%)	13423614 (39.87%)
Non-splice reads	16285571 (56.14%)	24639305 (56.15%)	19595841 (54.74%)	18366362 (55.55%)	14845008 (46.03%)	17260417 (48.82%)	17586663 (49.93%)	17013798 (50.53%)
Splice reads	6104196 (21.04%)	8962696 (20.43%)	7390118 (20.64%)	6607707 (19.98%)	9728004 (30.17%)	10544431 (29.82%)	10395480 (29.52%)	9800232 (29.1%)

f) Primer sequences used for qPCR

Gene ID	Symbol	Gene name	Primer sequences	Reference
102179831	ACTB	Actin beta	F: 5’TGCCCTGAGGCTCTCTTCCA<br>R: 5’TGCGGATGTCGACGTCACA	[1]
102169975	PRNP	Prion protein	F: 5’GTGGCTACATGCTGGGAAGT<br>R: 5’AGCCTGGGATTCTCTCTGGT	
102185230	IFI6	Interferon alpha inducible protein 6	F: 5’TATCGCTGTTCCTGTGCTACC<br>R: 5’AAGCTCGAGTCGCTGTTTTC	
100860873	CXCL10	C-X-C motif chemokine ligand 10	F: 5’ACGCTGTACCTGCATCGAG<br>R: 5’GCAGGATTGACTTGCAGGA	
102168428	SAA3	Serum amyloid A3	F: 5’CTGGGCTGCTAAAGTGATCAGTAAC<br>R: 5’CCCTTGAGCAGAGGGTCTGTGATT	[2]

1. Zhang Y, Zhang XD, Liu X, Li YS, Ding JP, Zhang XR, et al. Reference gene screening for analyzing gene expression across goat tissue. Asian-Australasian journal of animal sciences. 2013;26(12):1665-71.
2. Brenaut P, Lefèvre L, Rau A, Laloë D, Pisoni G, Moroni P, et al. Contribution of mammary epithelial cells to the immune response during early stages of a bacterial infection to Staphylococcus aureus. Vet Res. 2014;45:16.
