# Supplementary material for: LPS-induced systemic inflammation reveals an immunomodulatory role for the prion protein at the blood-brain interface
Source: J Neuroinflammation. 2017 May 22;14:106. doi: 10.1186/s12974-017-0879-5 (PMC5441080; doi:10.1186/s12974-017-0879-5)

**Additional file 2: Hematology and biochemistry**

**a) Hematology after day 1 LPS challenge (0.1 µg/kg).** EDTA blood tubes were collected before LPS challenge (0 h), and at 1 h, 5 h, and 24 h after LPS challenge. Figures show circulating levels of neutrophils, lymphocytes, monocytes and basophils at the indicated time points. *n* = 16

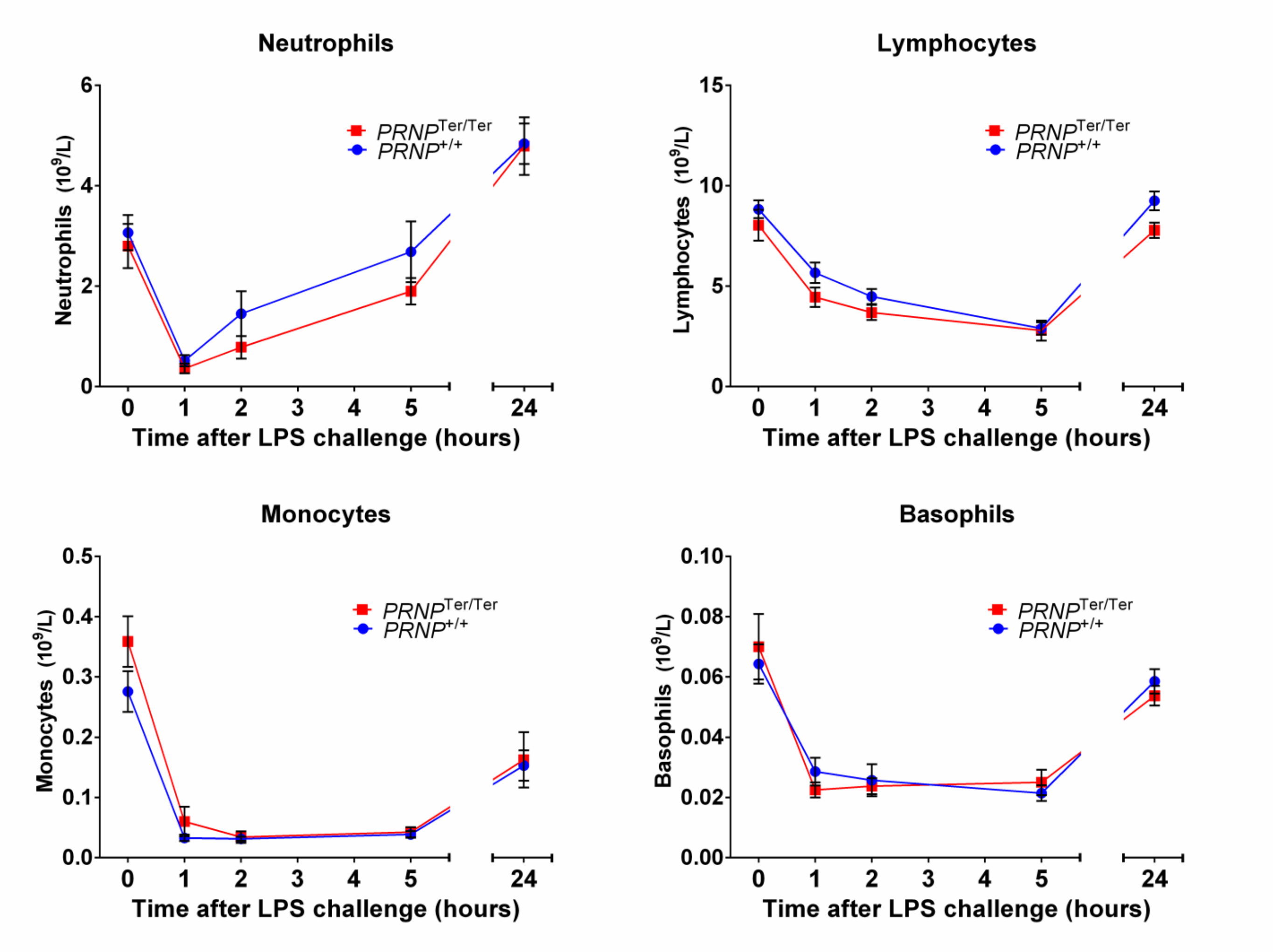

**b) Biochemical parameters after day 1 LPS challenge (0.1 μg/kg).** Serum was collected before LPS challenge (0 h), and at 1 h, 5 h, and 24 h after LPS challenge. *p<0.05; **p<0.01. *n* = 16

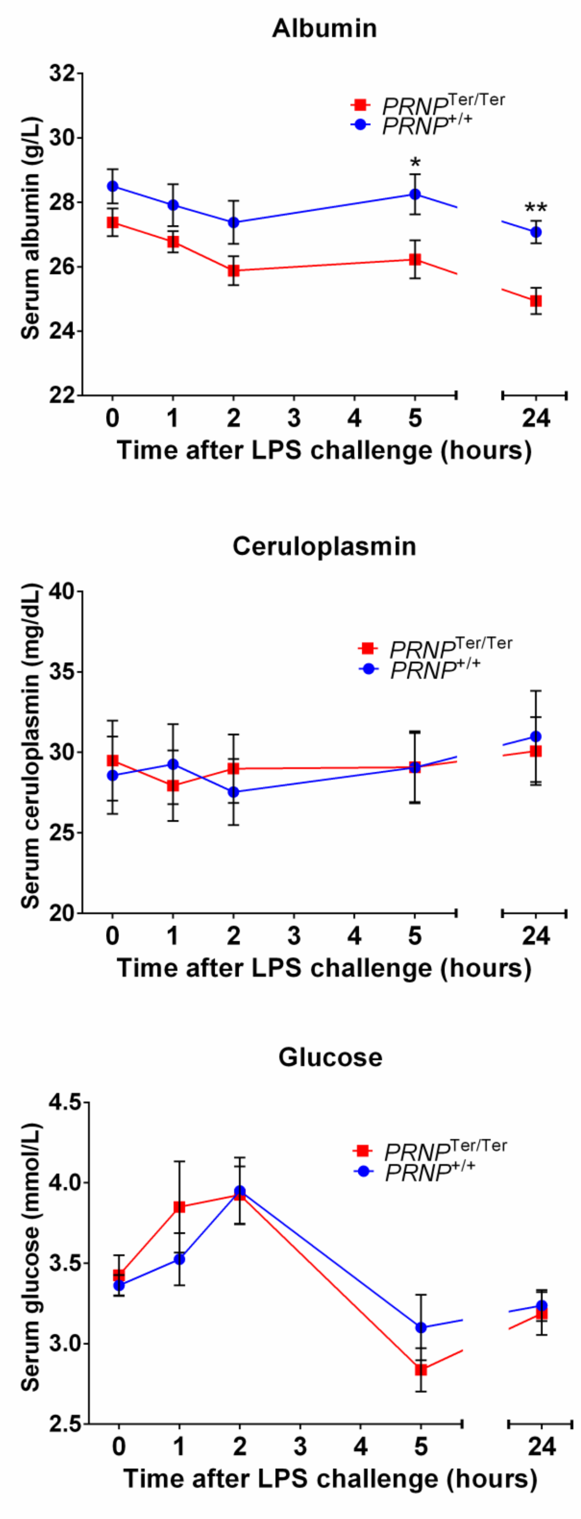

Supplement: Supplementary file 2 — Hematology and biochemistry after LPS challenge. (PDF 590 kb) [file 12974_2017_879_MOESM2_ESM.pdf]
