# Supplementary material for: LPS-induced systemic inflammation reveals an immunomodulatory role for the prion protein at the blood-brain interface
Source: J Neuroinflammation. 2017 May 22;14:106. doi: 10.1186/s12974-017-0879-5 (PMC5441080; doi:10.1186/s12974-017-0879-5)
Supplement: Supplementary file 3 — List of upregulated and downregulated genes in choroid plexus after LPS challenge. (PDF 458 kb) [file 12974_2017_879_MOESM3_ESM.pdf]

Additional file 3. Differentially expressed genes in the choroid plexus after LPS challenge.

a) Up-regulated genes in the choroid plexus after LPS challenge. Differential expression analyses were performed by comparing the LPS-groups to the saline control group of matching genotype. Bold values indicate a significant up-regulation (*log2 ratio >0.59, q<0.05). LPS, *n*=15. Saline, *n*=10

			Log2 ratio LPS vs. saline	
**Gene ID**	**Symbol**	**Gene name**	***PRNP*** $^{+/+}$	***PRNP*** $^{Ter/Ter}$
102187851	CXCL9	C-X-C motif chemokine ligand 9	**4,61***	**1,81***
100860873	CXCL10	C-X-C motif chemokine ligand 10	**4,46***	**3,24***
102174264	GBP6	Guanylate binding protein family member 6	**3,15***	**2,20***
100860781	SAA3	Serum amyloid A3	**3,13***	**3,14***
102188618	MT1A	Metallothionein-1A	**3,01***	**2,35***
102190797	RSAD2	Radical S-adenosyl methionine domain containing 2	**2,89***	**1,92***
102185230	IFI6	Interferon alpha inducible protein 6	**2,77***	**0,73***
102168687	IFIT1	Interferon-induced protein with tetratricopeptide repeats 1	**2,42***	**2,13***
102170076	SELP	Selectin P	**2,37***	**2,27***
102183943	CD163	Scavenger receptor cysteine-rich type 1 protein M130	**2,19***	**1,95***
106503920	APOL3	Apolipoprotein L3-like	**2,16***	**2,08***
102190722	OAS1	2’-5’-oligoadenylate synthase 1	**2,10***	**1,33***
102168428	SAA3	Serum amyloid A protein	**2,07***	**1,99***
102168968	IFIT3	Interferon induced protein with tetratricopeptide repeats 3	**2,00***	**1,73***
102185477	TGM2	Transglutaminase 2	**1,90***	**1,32***
102169982	ISG15	ISG15 ubiquitin-like modifier	**1,81***	**2,61***
102177056	CD14	CD14 molecule	**1,57***	**1,41***
102190983	OAS1L	2’-5’-oligoadenylate synthase 1-like	**1,52***	**1,77***
102170273	ICOSLG	Inducible T-cell 1ostimulatory ligand	**1,50***	**1,37***
102174491	LY6E	Lymphocyte antigen 6 complex, locus E	**1,42***	**1,23***
102188635	MX1	MX dynamin like GTPase 1	**1,39***	**1,89***
102180655	IFITM3	Interferon-induced transmembrane protein 3-like	**1,27***	**0,99***
102184389	MHCI	BOLA class I histocompatibility antigen, alpha chain BL3-6-like	**1,26***	**0,90***
102191085	COL6A1	Collagen type VI alpha 1 chain	**1,04***	**0,64***
102171079	COL4A1	Collagen type IV alpha 1 chain	**0,98***	**0,59***
102174427	RNF213	Ring finger protein 213	**0,73***	**0,79***
102191378	COL6A2	Collagen type VI alpha 2 chain	**0,73***	**0,68***
102173651	PLVAP	Plasmalemma vesicle associated protein	**0,65***	**0,61***
102170144	GBP	Guanylate-binding protein 1	**2,63***	1,64
102189747	HERC6	HECT and RLD domain containing E3 ubiquitin protein ligase family member 6	**2,59***	1,73
102176990	OAS3	2’-5’-oligoadenylate synthase 1/3	**2,49***	1,55
102169450	IFIT2	Interferon induced protein with tetratricopeptide repeats 2	**2,29***	1,57
102181449	PSMB9	Proteasome subunit beta 9	**2,20***	1,22
102184087	LGALS9	Galectin 9	**1,96***	1,16
102184922	GVIN1	Interferon-induced very large GTPase 1-like	**1,92***	1,21
102173932	IFI203	Interferon-activable protein 203-like	**1,88***	0,96
102180902	PSMB8	Proteasome subunit beta 8	**1,81***	0,93
102180584	MHCI	BOLA class I histocompatibility antigen, alpha chain BL3-7-like	**1,66***	0,47
102183942	CAPN6	Calpain 6	**1,63***	0,07
100860825	CHI3L1	Chitinase 3 like 1	**1,58***	1,46
102181173	TAP1	Transporter 1, ATP binding cassette subfamily B member	**1,55***	1,14
102168630	VCAN	Versican	**1,54***	1,47
102174541	IGFBP3	Insulin like growth factor binding protein 3	**1,37***	0,88
102173335	TNFSF10	Tumor necrosis factor superfamily member 10	**1,34***	0,89
102172487	CP	Ceruloplasmin (ferroxidase)	**1,32***	0,34
102188739	SRGN	Serglycin	**1,27***	1,02
102168722	ARHGAP26	Rho GTPase activating protein 26	**1,26***	0,67

102174895	VCAM	Vascular cell adhesion protein 1	**1,23***	0,94
102188814	MHCI	BOLA class I histocompatibility antigen, alpha chain BL3-7-like	**1,23***	0,12
102171143	APOL3	Apolipoprotein L3	**1,22***	1,20
102189585	SCARA5	Scavenger receptor class A member 5	**1,10***	0,52
102191086	COCH	Cochlin	**1,09***	-0,49
102177631	ADGRG6	Adhesion G protein-coupled receptor G6	**1,05***	-0,34
102178827	TPM2	Tropomyosin 2 (beta)	**1,04***	0,32
102181402	LCP1	Lymphocyte cytosolic protein 1	**1,01***	0,89
100860812	IGFBP4	Insulin like growth factor binding protein 4	**0,99***	0,28
102180712	DES	Desmin	**0,97***	0,57
102191633	DKK3	Dickkopf WNT signaling pathway inhibitor 3	**0,96***	-0,22
102172386	ADAMTS1	ADAM metallopeptidase with thrombospondin type 1 motif 1	**0,95***	0,43
102182694	CXCL14	C-X-C motif chemokine ligand 14	**0,93***	-0,09
102187830	COL17A1	Collagen type XVII alpha 1 chain	**0,83***	-0,53
102180259	C7	Complement C7	**0,82***	0,20
102169556	CXCL12	C-X-C motif chemokine ligand 12	**0,81***	-0,09
102191486	ACTA2	Actin, alpha 2, smooth muscle, aorta	**0,80***	0,30
102190641	MYLK	Myosin light chain kinase	**0,78***	0,23
102185937	TAGLN	Transgelin	**0,78***	0,37
102177315	FBLN1	Fibulin 1	**0,77***	-0,02
102188376	FGL2	Fibrinogen like 2	**0,75***	0,32
102172853	COL16A1	Collagen type XVI alpha 1 chain	**0,74***	-0,03
102177484	C4	Complement C4	**0,73***	0,08
102172761	ITGA1	Integrin subunit alpha 1	**0,73***	0,06
102176966	ACTN1	Actinin alpha 1	**0,71***	0,31
100860938	COL3A1	Collagen type III alpha 1 chain	**0,69***	-0,14
102179435	TPM3	Tropomyosin 3	**0,66***	0,29
100860940	COL1A2	Collagen type I alpha 2 chain	**0,65***	0,01
102188835	C1R	Complement C1r	**0,65***	0,22
102183179	GRN	Granulin	**0,65***	0,54
102175326	PFN1	Profilin 1	**0,63***	0,29
102180482	TPM4	Tropomyosin 4	**0,62***	0,51
102171347	COL4A2	Collagen type IV alpha 2 chain	**0,62***	0,22
102181355	PCOLCE	Procollagen C-endopeptidase enhancer	**0,61***	-0,10
102169304	ENG	Endoglin	**0,59***	0,24
102190143	KCTD12	Potassium channel tetramerization domain containing 12	**0,59***	-0,02
102174322	FOLR3	Folate receptor 3	1,11	**1,84***
102183788	DDX58	DEXD/H-box helicase 58	1,11	**1,62***
100861005	SLC29A1	Solute carrier family 29 member 1	0,94	**1,53***
102176743	UBA7	Ubiquitin like modifier activating enzyme 7	0,93	**1,09***
102186813	C1QB	Complement C1q B chain	0,92	**1,05***
102179622	DIRAS3	DIRAS family GTPase 3	0,85	**0,95***
102175447	FKBP5	FK506 binding protein 5	0,48	**0,79***
102177374	LGALS3BP	Galectin 3 binding protein	0,39	**0,78***
102168267	B2M	Beta-2-microglobulin	0,58	**0,68***

b) Down-regulated genes in the choroid plexus after LPS challenge. Differential expression analyses were performed by comparing the LPS-groups to the saline control group of matching genotype. Bold values indicate a true differential expression (*log2 ratio <0.59, q<0.05). LPS, *n*=15. Saline, *n*=10

			Log2 ratio LPS vs. saline	
**Gene ID**	**Symbol**	**Gene name**	***PRNP*** $^{+/+}$	***PRNP*** $^{Ter/Ter}$
102190015	PDK4	Pyruvate dehydrogenase kinase 4	**-1,47***	**-1,27***
102172168	GPR158	G protein-coupled receptor 158	**-0,66***	**-0,66***
102180778	RDH10	Retinol dehydrogenase 10 (all-trans)	**-2,23***	-0,51
102187800	UBB	Ubiquitin B	**-1,40***	0,30
102182855	TXNRD1	Thioredoxin reductase 1	**-0,78***	-0,36
102169722	TGFBR3	Transforming growth factor beta receptor 3	**-0,77***	0,10
102175994	TCAIM	T-cell activation inhibitor, mitochondrial	**-0,75***	-0,19
102172797	WNK1	WNK lysine deficient protein kinase 1	**-0,69***	-0,22
102177581	CACNA1H	Voltage-dependent T-type calcium channel subunit alpha-1H	**-0,67***	-0,38
102176607	SGK1	Serum/glucocorticoid regulated kinase 1	**-0,67***	-0,23
102187066	PPFIBP2	PPFIA binding protein 2	**-0,66***	-0,01
102183532	LRAT	Lecithin retinol acyltransferase	**-0,63***	-0,34
102184027	RBP4	Retinol binding protein 4	**-0,63***	-0,16
102189710	CLDN1	Claudin 1	**-0,63***	-0,03
102184132	METAP1	Methionyl aminopeptidase 1	**-0,62***	0,04
102176354	HP	Haptoglobin	-0,29	**-3,39***
102178465	ZBTB16	Zinc finger and BTB domain containing 16	-1,56	**-2,21***
102175527	CEMIP	Cell migration inducing hyaluronan binding protein	-0,06	**-1,41***
102189939	ATP5I	ATP synthase, H+ transporting, mitochondrial Fo complex subunit E	0,10	**-1,18***
106503375	C21H14orf132	Chromosome 21 C14orf132 homolog	-0,36	**-0,82***
102183476	ZYG11B	Zyg-11 family member B, cell cycle regulator	-0,21	**-0,73***
100860756	OGN	Osteoglycin	0,18	**-0,71***
102191385	VWA5B1	Von Willebrand factor A domain containing 5B1	-0,16	**-0,63***
