# Supplementary material for: LPS-induced systemic inflammation reveals an immunomodulatory role for the prion protein at the blood-brain interface
Source: J Neuroinflammation. 2017 May 22;14:106. doi: 10.1186/s12974-017-0879-5 (PMC5441080; doi:10.1186/s12974-017-0879-5)
Supplement: Supplementary file 4 — Gene ontology analyses of DEGs after LPS challenge. (PDF 312 kb) [file 12974_2017_879_MOESM4_ESM.pdf]

Additional file 4. Gene ontology analyses of differentially expressed genes (DEGs). The overall LPS effect was characterized by GO analyses of DEGs. The fold enrichment displays the over-representation of genes in a given biological process, compared with the expected number in the reference genome (*Homo sapiens*). P-value <0.05 represents a statistically significant over-representation and is calculated by the binomial test.

a) GO over-representation test of 92 up-regulated genes in the choroid plexus after LPS challenge.

GO biological process	Genes	Gene names	Fold	P-value
Type I interferon signaling pathway (GO:0060337)	11	IFITM3, IFIT1, IFIT2, IFIT3, OAS1, OAS3, PSMB8, MX1, RSAD2, IFI6, ISG15	45.2	1.49E-11
Positive regulation of leukocyte migration (GO:0002687)	7	CXCL9, CXCL10, CXCL12, CXCL14, LGALS9, SELP, PLVAP	15.5	0.0034
Extracellular matrix organization (GO:0030198)	12	COL1A2, COL3A1, COL4A1, COL4A2, COL6A1, COL6A2, COL16A1, ITGA1, FBLN1, VCAN, LCP1, ENG	10.0	2.66E-05
Defense response to other organism (GO:0098542)	17	IFITM3, DDX58, CXCL9, MYLK, CXCL10, COCH, B2M, IFIT2, SELP, IFIT3, MX1, OAS1, RSAD2, IFIT1, ISG15, OAS3, GBP6	8.8	6.88E-08
Response to cytokine (GO:0034097)	23	CXCL9, CXCL10, CD14, B2M, PSMB9, LGALS9, TGM2, IFIT2, IFIT3, PLVAP, CHI3L1, PSMB8, IFITM3, MX1, OAS1, CXCL12, RSAD2, IFIT1, COL3A1, IFI6, ISG15, OAS3, GBP6	7.9	5.63E-11
Innate immune response (GO:0045087)	19	DDX58, CD14, B2M, C7, C1QB, LGALS9, IFIT2, C1R, IFIT3, PSMB8, MX1, OAS1, RSAD2, IFIT1, IFI6, ISG15, IFITM3, OAS3, GBP6	7.9	2.01E-08

b) GO over-representation test of 10 up-regulated genes in the hippocampus after LPS challenge.

GO biological process	Genes	Gene names	Fold	P-value
Cytokine-mediated signaling pathway (GO:0019221)	5	MT2, CXCL9, CXCL10, TGM2, IFI6	22.5	0.010

c) GO over-representation test of 25 down-regulated genes in the choroid plexus after LPS challenge.

GO biological process	Genes	Gene names	Fold	P-value
Cellular hormone metabolic process (GO:0034754)	5	LRAT, RBP4, RDH10, CACNA1H, UGT1A1	40.3	0.016
Retinoid metabolic process (GO:0001523)	4	LRAT, RBP4, RDH10, UGT1A1	37.7	0.032
Regulation of biological quality (GO:0065008)	15	WNK1, SGK1, METAP1, LRAT, CEMIP, RBP4, RDH10, CLDN1, TXNRD1, PDK4, CACNA1H, UGT1A1, UBB, FGF10, TGFBR3	3.5	0.013
