# Supplementary material for: LPS-induced systemic inflammation reveals an immunomodulatory role for the prion protein at the blood-brain interface
Source: J Neuroinflammation. 2017 May 22;14:106. doi: 10.1186/s12974-017-0879-5 (PMC5441080; doi:10.1186/s12974-017-0879-5)

Additional file 5: Validation of RNA-seq data by qPCR. Comparison of relative mRNA expression detected by RNAseq and qPCR. For each gene and tissue, expression levels are relative to the saline-treated *PRNP*$^{+/+}$ group (=1). For RNAseq, symbols represent a significant differential expression (log2 ratio ± 0.59, q<0.05). qPCR bars display mean expression ±SEM with significance levels of p<0.05, p<0.01 p<0.001 (Student's t-test). Symbols are listed below. In the hippocampus, SAA3 was not detected at rest by RNAseq and at very low levels after LPS challenge.

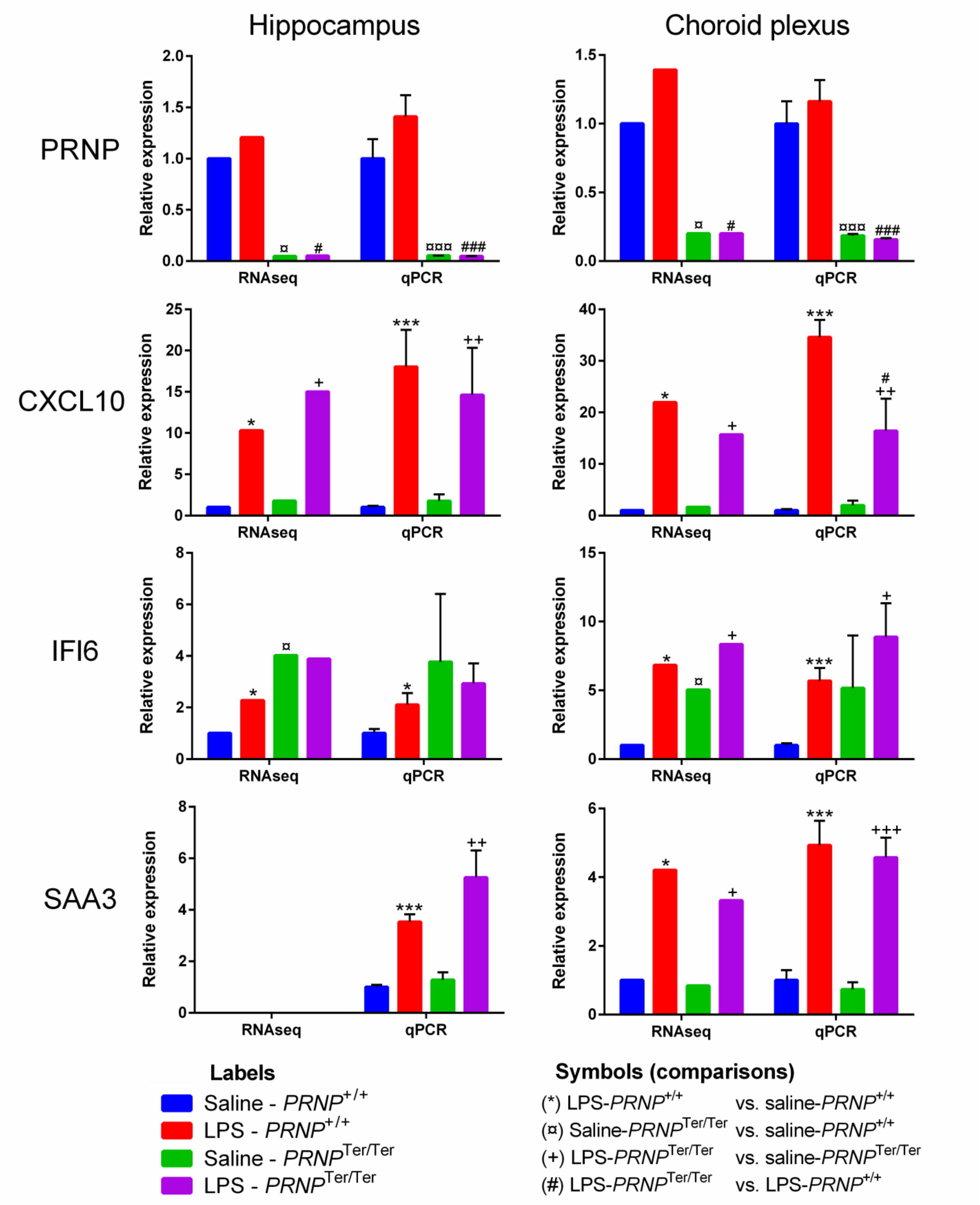

Supplement: Supplementary file 5 — Validation of RNAseq data by qPCR. (PDF 484 kb) [file 12974_2017_879_MOESM5_ESM.pdf]
